# A Comparative Study of the Sintering Behavior of Pure and Manganese-Substituted Hydroxyapatite

**DOI:** 10.3390/ma8095308

**Published:** 2015-09-18

**Authors:** Michael Zilm, Seamus D. Thomson, Mei Wei

**Affiliations:** 1 Department of Materials Science and Engineering, University of Connecticut, 97 North Eagleville Rd, Unit 3136, Storrs, CT 06269, USA; E-Mail: michael.zilm@uconn.edu; 2 Department of Aerospace, Mechanical and Mechatronic Engineering, J07 University of Sydney, University of Sydney, Sydney, NSW 2006, Australia; E-Mail: stho6915@uni.sydney.edu.au

**Keywords:** hydroxyapatite, ion substitution, sintering behavior

## Abstract

Hydroxyapatite (HA) is a widely studied biomaterial for its similar chemical composition to bone and its osteoconductive properties. The crystal structure of HA is flexible, allowing for a wide range of substitutions which can alter bioactivity, biodegradation, and mechanical properties of the substituted apatite. The thermal stability of a substituted apatite is an indication of its biodegradation *in vivo*. In this study, we investigated the thermal stability and mechanical properties of manganese-substituted hydroxyapatite (MnHA) as it is reported that manganese can enhance cell attachment compared to pure HA. Pure HA and MnHA pellets were sintered over the following temperature ranges: 900 to 1300 °C and 700 to 1300 °C respectively. The sintered pellets were characterized via density measurements, mechanical testing, X-ray diffraction, and field emission electron microscopy. It was found that MnHA was less stable than HA decomposing around 800 °C compared to 1200 °C for HA. The flexural strength of MnHA was weaker than HA due to the decomposition of MnHA at a significantly lower temperature of 800 °C compared to 1100 °C for HA. The low thermal stability of MnHA suggests that a faster *in vivo* dissolution rate compared to pure HA is expected.

## 1. Introduction

Hydroxyapatite (HA) is an inorganic constituent of natural bone, which has been extensively studied for its biocompatible and osteo-regenerative properties, serving a scaffold for modern bone graft substitutes [1,2,3]. As such, HA is widely used for coating orthopedic implants where a strong interface with bone is required and as a bone cement for craniofacial repair [4,5]. However, HA is brittle in nature and has a slow *in vivo* degradation rate which limits its applications to coatings for orthopedic implants [2,6,7,8]. To overcome these issues, HA may be densified through sintering to improve mechanical strength and or through ionic substitutions to enhance bioactivity and mechanical strength. [7,8,9,10,11,12,13,14,15].

The pressing and sintering of HA have been known to improve its ultimate compressive strength and toughness [7,15]. Sintering can occur as low as 800 °C with the final sintered density serving as a function of the particle size distribution and agglomeration characteristics of the starting HA powder [9,10]. For temperatures greater than 700 °C, HA begins to decompose into different phases such as tricalcium phosphate (TCP), calcium oxide (CaO) and water (H_2_O) [16]. Overall, the presence of TCP in HA scaffolds affects the strength, density, pore size, and degradation rate of HA [15,17]. TCP degrades faster than HA *in vivo*.

Stoichiometric HA has been generally accepted as having a hexagonal P6_3_/m structure with chemical formula Ca_10_(PO_4_)_6_(OH)_2_ to denote the chemical composition and symmetry composing the crystal unit cell [16,18]. This apatite structure is flexible, which can be substituted by various elements, such as Na, Mg, K, Sr, Zn, Ba, Cu, Al, Fe, F, Cl, and carbonate ions [11,13,15]. Alteration of the chemical formula of HA through substitutions can alter its bioactivity, which is a similar approach to modify the bioactivity of bioglasses [8].

In improving the bioactivity of a material it is critical for the material to support cell attachment and the extracellular matrix which contains bioactive factors to induce regeneration [19]. When manganese is substituted into the HA lattice (MnHA) the MnHA has the attractive feature of improving cellular adhesion [20,21,22,23,24]. Manganese (Mn) is an essential trace metal found in all tissues and is required for normal amino acid, lipid, protein, and carbohydrate metabolism [25,26]. Mn plays a crucial role in cellular signaling, especially in the activation of integrins; receptors that mediate cellular interactions between the extracellular matrix and ligands on cell surfaces [12,27]. The ligand affinity increases for integrins in the presence of Mn ions, promoting cellular adhesion and possibly having the ability to enhance osteogenesis [27,28]. The development of new tissue engineered scaffolds that provide mechanical support as well as repair and regeneration of damaged or diseased bone may readily benefit from the introduction of Mn into current HA scaffolds [2].

MnHA has been previously synthesized through various wet synthesis techniques [12,28,29,30,31,32,33,34,35,36,37,38]. Many of these studies examine the stability of MnHA through varying the wt % of manganese or the calcination temperature. Mayer *et al.* attempted to incorporate manganese up to 6 wt % via wet synthesis which decomposed at 600 °C into β-TCP [12,34,35]. Other attempts to incorporate up to 5 wt % manganese followed by calcination at 800 °C, by Paluszkiewicz *et al.* resulted in phase purity up to 1 wt % manganese [36]. The phase stability of MnHA at varying manganese wt % is well understood but there are few studies on the effect of manganese substitution and mechanical properties. Ramesh *et al.* produced MnHA through a wet milling technique and observed a decrease in the Vickers hardness of sintered MnHA up to 1 wt % relate density [39]. Herein we report a facile one-step room temperature ion exchange method to synthesize MnHA, as well as a systematic study of the sintering behavior, microstructural evolution and flexural strength over a series of temperatures to determine the suitability of MnHA for biomedical applications. The relationship between mechanical properties, microstructure and phases helps identify suitable biomedical applications for pure MnHA and its decomposition products.

## 2. Results and Discussion

### 2.1. Results

#### 2.1.1. Powder Characterization

**Figure 1:**
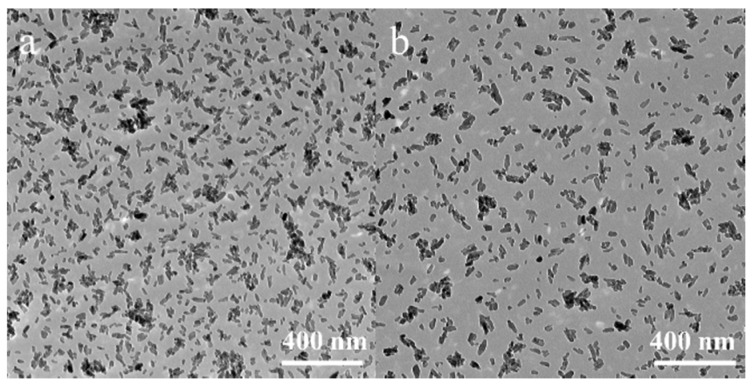
TEM micrographs of SPEX-milled Hydroxyapatite (HA) (**a**) and manganese-substituted hydroxyapatite (MnHA) (**b**) powders.

SPEX-milled as-synthesized HA and MnHA powders were characterized with TEM for particle size analysis. Micrographs of HA and MnHA powders are depicted in Figure 1. Both powders have morphologies of small rods with an average aspect ratio of 2.6 ± 0.8 for HA and 2.4 ± 0.7 for MnHA particles. HA particles (*n* = 151) have an average length of 35 ± 11 nm and an average width of 14 ± 3 nm. Similar to HA, MnHA particles (*n* = 155) have an average length of 36 ± 11 nm and a width of 16 ± 4 nm.

#### 2.1.2. XRD Characterization

**Figure 2:**
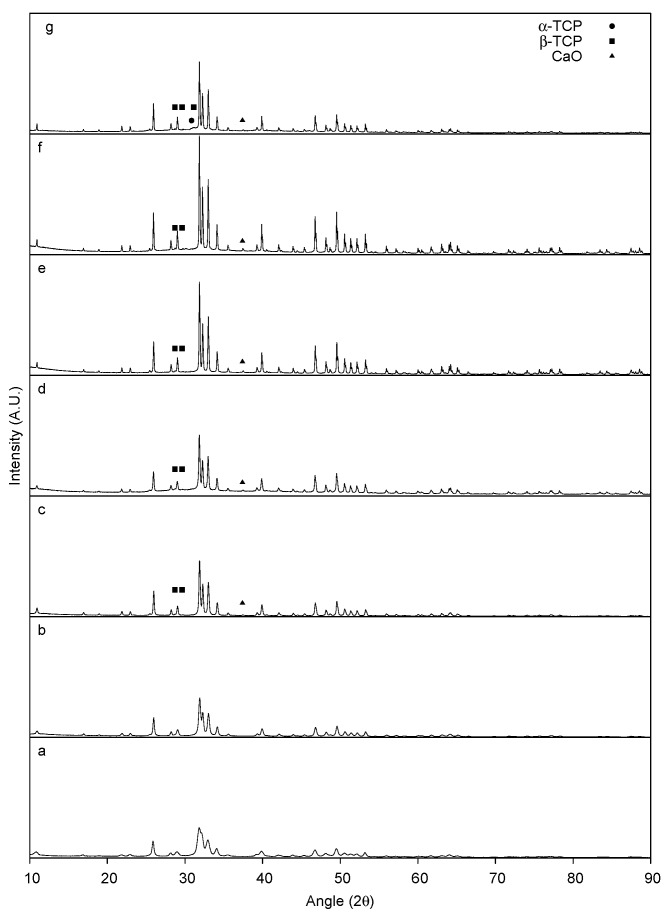
Powder XRD pattern of as-synthesized HA (**a**) and HA heat treated at: 800 °C (**b**); 900 °C (**c**); 1000 °C (**d**); 1100 °C (**e**); 1200 °C (**f**); and 1300 °C (**g**). Non-labeled peaks correspond to observed reflections from pure HA.

Powder diffraction patterns of the as-synthesized and heat-treated HA are depicted in Figure 2. The as-synthesized material displays broad peaks and is identified as HA based on JCPDF 9-432. At 800 °C, the observed peaks become more intense with no extraneous peaks. The indexed peaks for HA continue to increase in intensity up to 1200 °C and decrease at 1300 °C. Decomposition of HA is observed around 900 °C with the emergence of calcium oxide (CaO) and β-tricalcium phosphate (β-TCP) peaks indexed at 27.8° and 29.68° 2θ for β-TCP and 37.4° for CaO. At 1300 °C, HA decomposes further and α-TCP peaks started to emerge.

**Figure 3:**
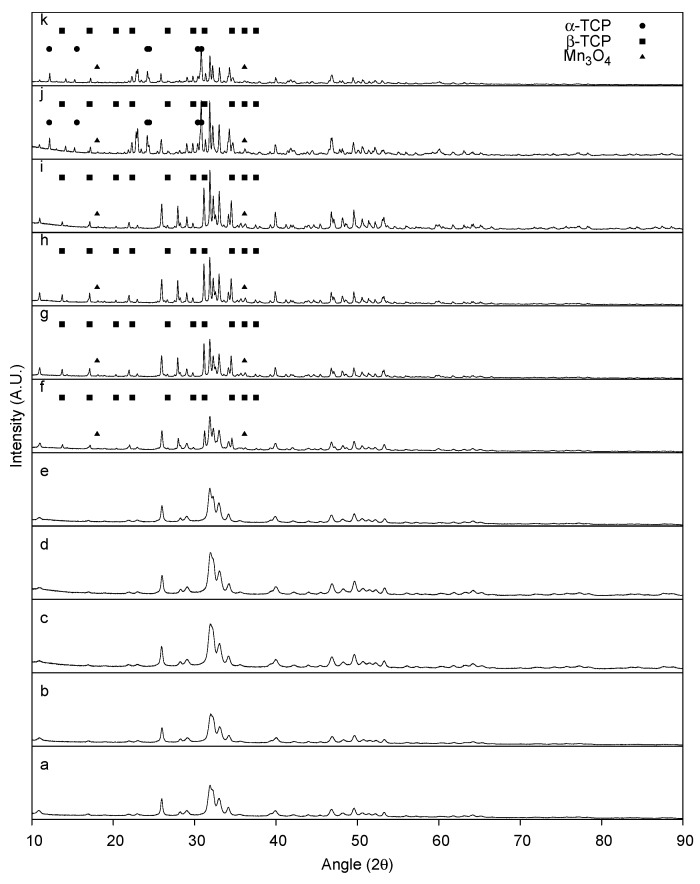
Powder XRD pattern of as-synthesized MnHA (**a**) and MnHA heat-treated at: 400 °C (**b**); 500 °C (**c**); 600 °C (**d**); 700 °C (**e**); 800 °C (**f**); 900 °C (**g**); 1000 °C (**h**); 1100 °C (**i**); 1200 °C (**j**); and 1300 °C (**k**). Non-labeled peaks correspond to observed reflections from pure HA.

In comparison to the as-synthesized HA, the as-synthesized MnHA exhibits the same powder diffraction pattern indicative of a pure Mn substituted HA, as shown in Figure 3. Unlike HA, MnHA start to decompose to β-TCP and manganese oxide (Mn_3_O_4_) at a temperature as low as 800 °C, Figure 3, with continuous decomposition at higher temperatures. At 1200 °C further decomposition into α-TCP is observed producing a mixture of α-TCP, β-TCP, and Mn_3_O_4_.

#### 2.1.3. Density and Biaxial Flexural Strength

**Figure 4:**
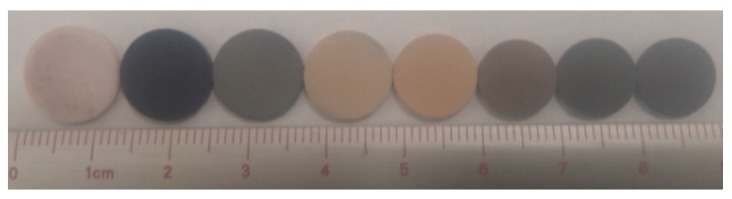
Photograph of green and sintered MnHA pellets. From left to right: green MnHA followed by MnHA heat-treated at 700–1300 °C at 100 °C increments. The pellet size also decreases from left to right.

After sintering but prior to density measurements and mechanical testing the physical appearance of the sintered pellets was observed. Sintered HA pellets underwent shrinkage at all temperatures but maintained their white appearance. In contrast, the MnHA pellets not only underwent shrinkage but also resulted in a change in physical appearance which is depicted in Figure 4. The initial green pellet is light pink but after sintering at 700 °C turns dark blue. Subsequent sintering temperatures resulted in further color transitions with gray pellets observed at 800 °C, yellow from 900 to 1000 °C, reddish brown at 1100 °C, and black from 1200 to 1300 °C.

**Figure 5:**
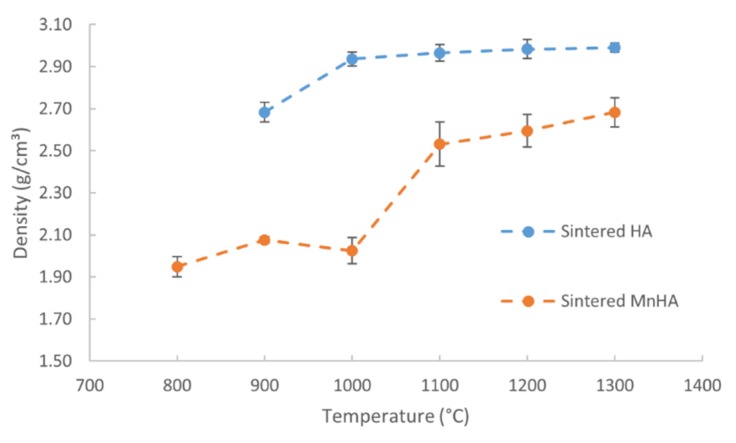
Densities of sintered HA and MnHA pellets as a function of temperature. Average densities are plotted ± standard deviation (*n* ≥ 4).

The initial green densities, after cold isostatic pressing, and final sintered densities are summarized in Table 1 along with the percent densified. Green bodies of HA have an average density around 1.8 g/cm^3^, while MnHA have a density around 1.65 g/cm^3^. The final densities of HA and MnHA pellets at various soaking temperatures are depicted in Figure 5. A one way ANOVA analysis was performed to determine statistically significant differences. There was a statistically significant difference (*p* < 0.05) in HA densities (*p* = 5.93 × 10^−16^) and MnHA densities (*p* = 5.38 × 10^−35^) over the studied temperature ranges. After sintering at 900 °C the HA pellets start to densify followed by increased densification upon sintering at 1000 °C. Within the temperature range of 1000 to 1300 °C, HA pellets continue to densify with increasing temperature until 1300 °C, where a maximum density of 2.99 g/cm^3^ is achieved. For MnHA specimens the sintered density continuously increases over the studied temperature range which is reflected in the percent densified. An abrupt increase in density is observed from 900 to 1000 °C corresponding to a density of 2.02 and 2.53 g/cm^3^, respectively. From 1000 to 1300 °C the density continues to increases and reaches a maximum density of 2.68 g/cm^3^ at 1300 °C.

**Table 1:** Average density values of green and sintered bodies and percent densification for each studied temperature point.

Temperature (°C)	Average Green Density (g/cm^3^)		Average Sintered Density (g/cm^3^)		Average Percent Densified	
	HA	MnHA	HA	MnHA	HA	MnHA
800	–	1.78	–	1.95	–	9.4
900	1.80	1.76	2.68	2.08	49.4	18.2
1000	1.81	1.58	2.94	2.02	62.0	28.1
1100	1.83	1.62	2.97	2.53	61.9	56.4
1200	1.80	1.59	2.98	2.59	65.4	62.9
1300	1.81	1.58	2.99	2.68	65.5	70.2

**Figure 6:**
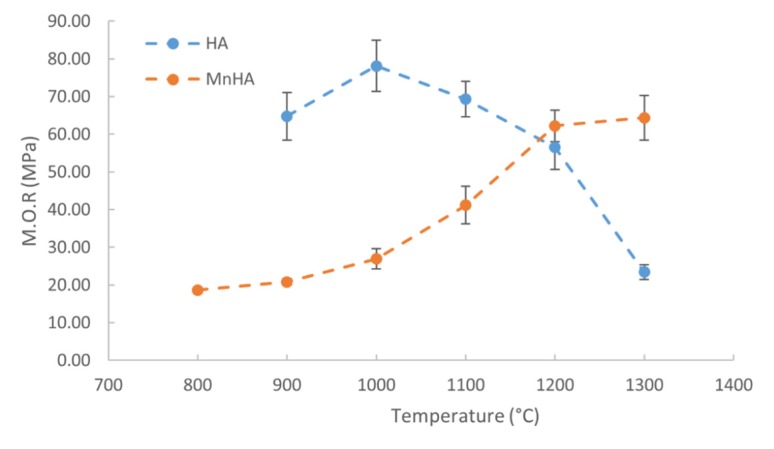
Mechanical properties of sintered HA and MnHA pellets as a function of temperature. Average M.O.R values are plotted ± standard error (*n* ≥ 4).

Due to the compressive and bending forces typically exerted on bones, the flexural strength of the sintered pellets were evaluated by a modulus of rupture (M.O.R) test, and the mechanical properties are depicted in Figure 6. A one way ANOVA was performed to determine statistically significant differences. There was a statistically significant difference (*p* < 0.05) in M.O.R between HA (*p =* 1.33 × 10^−7^) and MnHA (*p* = 4.4 × 10^−13^) over the studied temperature ranges. The M.O.R of sintered HA pellets ranges from 23 MPa at 1300 °C to 78 MPa at 1000 °C. Initially the M.O.R increases from 900 to 1000 °C and then continuously decreases from 1000 to 1300 °C. Unlike HA, the M.O.R. values of the sintered MnHA continuously increase over the temperature range of 800 to 1300 °C ranging from 18 MPa at 800 °C to 64 MPa at 1300 °C. From 800 to 1000 °C there is a small gradual increase in the M.O.R values followed by a rapid increase over the range of 1000 to 1200 °C with a small increase from 1200 to 1300 °C.

#### 2.1.4. FESEM Characterization

**Figure 7:**
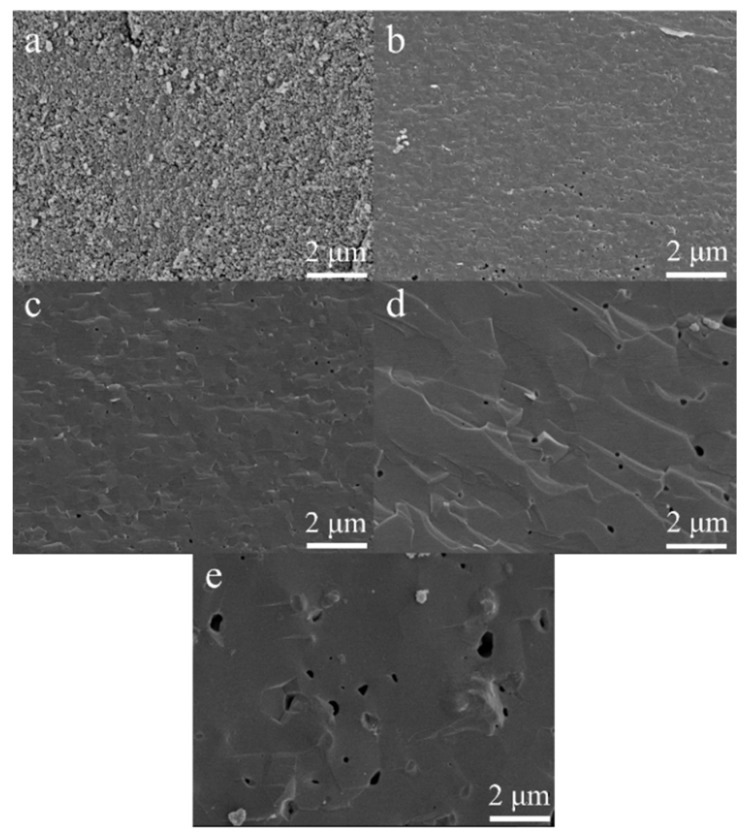
Cross sections of sintered HA pellets at 900 °C (**a**); 1000 °C (**b**); 1100 °C (**c**); 1200 °C (**d**); and 1300 °C (**e**). Micrographs (**a**–**e**) were taken at 10,000 × magnification.

The sintering behavior of heat-treated HA and MnHA specimens was further investigated using FESEM. Fractured surfaces of sintered HA pellets are depicted in Figure 7. Specimens heat treated at 900 °C exhibit signs of sintering where the HA particles have fused together and the pores between particles have started to round, but there are still a large number of pores present in specimens. At 1000 °C, the HA specimens have sintered completely with few rounded pores present. At temperatures greater than 1000 °C, grain growth is observed with increasing temperature. Pores remain over the range of 1000 to 1300 °C with a rounded morphology and grow in size with increasing temperature.

**Figure 8:**
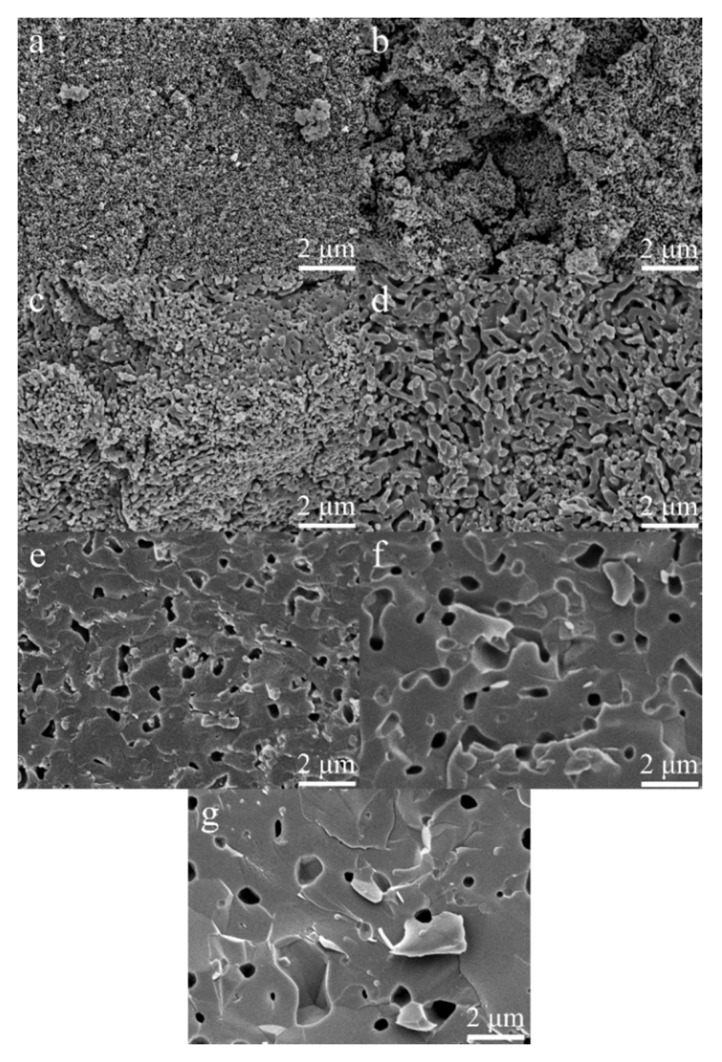
Cross sections of sintered MnHA pellets at 700 °C (**a**); 800 °C (**b**); 900 °C (**c**); 1000 °C (**d**); 1100 °C (**e**); 1200 °C (**f**); and 1300 °C (**g**). Micrographs (**a**–**g**) were taken at 10,000× magnification.

Fracture surfaces of heat-treated MnHA pellets are depicted in Figure 8. At 700 °C the particles of the green body are identifiable and there is no indication of particles fusing at this temperature. Unlike HA, MnHA starts to sinter at a temperature as low as 800 °C, where individual particles have started to fuse together, but the overall structure still demonstrates a porous network. The onset of decomposition of MnHA also occurred at 800 °C. The pellets continue to sinter with increasing temperature and the porosity of the sintered body slowly decreases. Significant porosity increase is present at 1200 °C, which is 100 °C lower than that observed in HA sintering.

**Figure 9:**
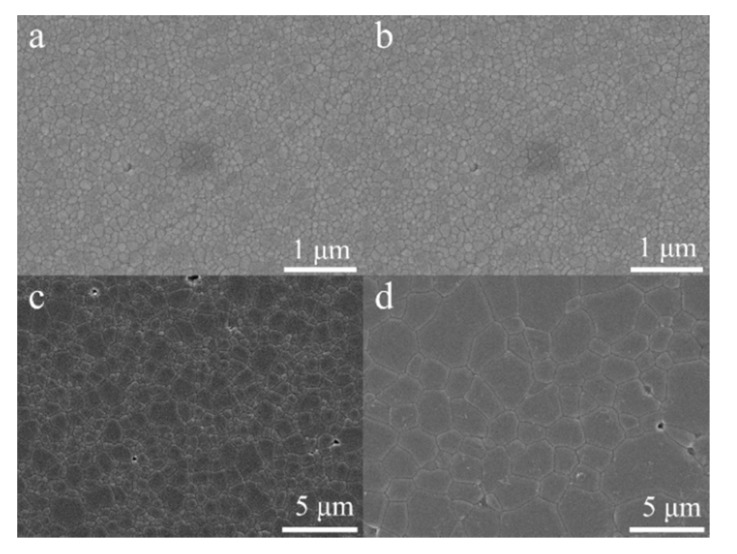
FESEM images of the etched surface of HA pellets sintered at 1000 °C (**a**); 1100 °C (**b**); 1200 °C (**c**); and 1300 °C (**d**). Note the differences in magnification. Micrographs (**a**) and (**b**) were taken at 25,000×. Micrographs (**c**) and (**d**) were taken at 5000×.

**Figure 10:**
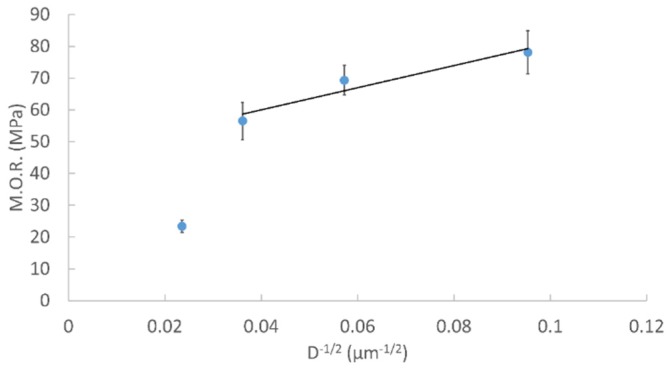
Modulus of rupture of HA as a function of the inverse square root of the grain size over the temperature range from 1000 to 1300 °C.

Since the sintering of HA specimens was complete at 1000 °C, FESEM was used to for grain size analysis over the temperature range from 1000 to 1300 °C. Figure 9 shows polished and etched surfaces of HA sintered bodies. The measured grain size was 110, 305, 765 nm, and 1.8 μm from 1000 to 1300 °C. A relationship between the M.O.R and the inverse square root of grain size of sintered HA bodies is depicted in Figure 10. The M.O.R increases with decreasing grain size following a linear relationship from 1000 to 1200 °C, which follows the classical Hall-Petch relationship with the following relation

(1) $$M.O.R=\tau_{o}+kd^{-\frac{1}{2}}$$

where τ_o_ and *k* are material constants and *d* is the average grain size.

### 2.2. Discussion

The ceramic phase of bone is a poorly crystalline phase of HA with numerous impurities which influence HA’s thermal stability, and mechanical and bioactive properties. Pure HA is normally stable until 1350 °C before decomposing into calcium oxide (CaO) and tricalcium phosphate (TCP) [40]. In the current study, the decomposition of the as-synthesized HA into CaO and β-TCP started at 900 °C. HA with chemical impurities, such as carbonate, have been shown to degrade at temperatures as low as 800 °C [41]. The synthesis method used in this study was exposed to an ambient atmosphere which resulted in partial carbonation of the precipitated HA, as previously reported [42]. As the temperature increases from 900 to 1200 °C, the HA phase increases in crystallinity with temperature. A transition is observed at 1300 °C, where the peak intensity of the HA phase decreases in intensity compared to 1200 °C. The relative intensity between the decomposed phases β-TCP and CaO to HA is greater at 1300 °C compared to 1200 °C indicating that the decomposition of the HA phase increases with the sintering temperature.

The thermal stability of MnHA was investigated over the range of 400 to 1300 °C. A larger temperature range than HA was selected since manganese oxides have been shown to undergo phase transitions as low as 400 °C [43]. The as-synthesized MnHA powder diffraction pattern matches the observed pattern for the as-synthesized HA indicating successful substitution of Mn into the HA lattice. The phase purity of MnHA is maintained up to 800 °C where significant decomposition into β-TCP and Mn_3_O_4_ has occurred. Upon further heating, MnHA decomposes into α-TCP, β-TCP, and Mn_3_O_4_ at 1200 °C; these phases remain present at 1300 °C. The transformation of β-TCP to α-TCP is typically observed around 1125 °C [44].

The microstructure of HA and MnHA were investigated with FESEM over the temperature range studied for mechanical testing. Pure HA normally starts to sinter around 1000 °C [40], but in this study HA was observed to sinter at 900 °C. HA nanorods with similar reported aspect ratios around 2.4 and an average length around 55 nm have been shown to start to sinter as low as 850 °C [45]. The sintering behavior at low temperatures resulted from surface defects such as OH^−^ vacancies and vacancies created from the release of carbonate groups which lowers the activation energy for surface diffusion [45]. Proceeding from 900 to 1000 °C sintering has completed which is observed in the closure of the pores between particles and an increase in the sintered density which plateaus around 95% of the theoretical density (3.156 g/cm^3^) over the rest of the studied temperature range. The grain size and pore size increase with the sintering temperature. The grains grow as part of the recrystallization and growth process, while the increase in pore size is a result of the dehydroxylation process of HA. At temperatures exceeding 800 °C HA starts to lose water; during this process pressure builds from water vapor and results in blowhole formation with larger blowholes at higher temperatures.

In the case of MnHA sintering occurred around 800 °C, which coincided with the onset of decomposition. The decomposition of MnHA over the temperature range of 800 to 1000 °C results in a highly porous and interconnected structure which may be attributed to the counteracting processes of both sintering and dehyrdoxylation of HA. The overall porosity slowly decreases over this temperature range. The porosity significantly decreases at 1100 °C, which is reflected in the increase in densification. The pores have become isolated and are lager in size. A similar microstructure was observed at 1200 and 1300 °C, but with less porosity and larger pores. The porosity over the range of 1100 to 1300 °C is related to the decomposition products of HA.

Interestingly over this temperature range the sintered densities increased slowly as seen in the percent densification, and the higher sintered density at 900 °C compared to 1000 °C is attributed to the greater initial green density of 900 °C specimens compared to 1000 °C. The differences between these green densities were a result of variation in batch to batch processing of MnHA where the degree of agglomeration influences the final green density after pressing.

HA and MnHA display different densification and mechanical behavior as a function of temperature. The difference in densification behavior is a result of their discrepancy in thermal stability which influences the microstructure as discussed above. Meanwhile, the mechanical behavior is closely related to the microstructure of the material. HA pellets achieved a maximum M.O.R around 78 MPa, which compares favorably with the values reported in literature of bulk HA which range from 45 to 150 MPa [7,46,47,48,49,50,51,52,53,54]. The wide range of reported values results from differences in processing and impurities. Hot isostatic pressing which results in a more homogenous microstructure and fewer defects was used by Boilet *et al.* and Raynaud *et al.* to achieve flexural strengths in excess of 100 MPa [53,54]. Kana *et al.* observed that carbonated HA has a lower flexural strength, 45 MPa, when compared to pure HA, 75 MPa [51]. The literature values of flexural strength and the observed maximum M.O.R are also within the range of reported flexural moduli of natural bone which ranges from 35 to 283 MPa [55].

The M.O.R shown in Figure 6 depicts that the M.O.R initially increases for HA peaking at 1000 °C and then decreases over the rest of the temperature range. The initial increase is a result of fewer pores from improved densification from 900 to 1000 °C. After 1000 °C the M.O.R continuously decreases with increasing temperature. Three mechanisms may be attributed to the decreased M.O.R: (1) larger grains; (2) higher porosity; and (3) more decomposition. Improved strength and hardness are observed in ceramics with smaller grains which provide more barriers to crack propagation compared to larger grains. Pores are sites of concentrated stress which reduces the stress need for crack initiation and propagation leading to premature failure. The decomposition process of HA results in pore formation and inhomogeneous microstructure which also reduce the overall strength. The main mechanism resulting in a decrease in the M.O.R is an increase in grain size from 1000 to 1200 °C which follows the classical Hall-Petch relation. A similar Hall-Petch relation was observed in the hardness of nanocrystalline HA sintered over the temperature range of 850 to 1200 °C with grain sizes ranging from 65 to 730 nm by Wang *et al.* [56]. At 1300 °C the M.O.R deviates from the Hall-Petch relation as a result of HA decomposition to TCP. The reduction in M.O.R is a result of increased grain size, porosity and pore size.

The increase in the M.O.R as a function of temperature for MnHA is attributed to the sintering behavior of the material, as MnHA densifies the degree of porosity decreases. At sintering temperatures lower than 1100 °C, a highly porous and interconnected microstructure is observed. The large degree of porosity and the interconnectivity of the pores result in a fragile structure. The formation of a denser microstructure with isolated pores observed from 1100 to 1300 °C results in an increase in the M.O.R attributed to a reduction in porosity. The maximum M.O.R observed for MnHA is around 64 MPa at 1300 °C which is lower than the maximum M.O.R observed for pure HA. Sintering at 1200 °C, the HA phase has almost completely decomposed with α-TCP, β-TCP, and Mn_3_O_4_ being the dominate phases. The flexural strength of β-TCP is weaker than natural bone and is often detrimental to the mechanical properties of HA when presents as an impurity [53,57]. The maximum observed M.O.R of MnHA of 64 MPa compares favorably with literature values. Studies on the mechanical properties of metal substituted β-TCP and sintered laminate β-TCP have shown flexural strengths ranging from 20 to 160 MPa [58,59]. Despite the decomposition of MnHA to TCP, the fast degradation rate of TCP sometimes is a desirable property in many biomedical applications. TCP has been mixed with HA to produce tissue engineering scaffolds with accelerated degradation rate compared to pure HA scaffolds [60].

## 3. Experimental Section

### 3.1. Materials

The following materials were purchased from Acros Organics (Grand Island, NY, USA): Ammonium phosphate dibasic (99+%), sulfuric acid (95%–98%), stearic acid (97%), and potassium bromide (IR grade). Calcium nitrate (99+%), manganese chloride tetrahydrate (99+%), and ammonium hydroxide (29.45%) were purchased from Fisher Scientific (Pittsburh, PA, USA).

### 3.2. Hydroxyapatite and Manganese Hydroxyapatite Synthesis

HA was synthesized via a wet chemical method. An aqueous solution of ammonium phosphate dibasic (80 mM) was added drop-wise to an aqueous calcium nitrate solution (225 mM) at room temperature with the calcium to phosphate ratio maintained at 1.667. Ammonium hydroxide was used to raise the pH of both solutions above 11 prior to mixing the two solutions. Once the two solutions were completely mixed, the temperature of the solution was raised to 95 °C for five hours, and then it was cooled to room temperature and aged for two days under constant stirring. The precipitates were collected via centrifugation and washed 3 times with de-ionized, 2 times with ethanol and vacuumed dried.

MnHA was synthesized through an ion-exchange procedure. A salt solution of MnCl_2_ was prepared at a concentration of 0.02 M and the pH was adjusted to 2.7 with dilute sulfuric acid. Previously synthesized HA was immersed in the ion-exchange solution at 0.5 g/dL and sonicated for one hour at room temperature with intermittent stirring. The ion-exchanged HA was collected by centrifugation, washed 3 times with DIW and then vacuumed dried.

The collected precipitates were ground with a mortar and pestle for 30 min into a fine powder. The resulting powders were then milled (SPEX 8000 Mixer/Mill, SPEX, Metuchen, NJ, USA) for 10 min in a steel canister with alumina mixing balls.

### 3.3. Pellet Preparation and Sintering

SPEX-milled HA and MnHA powders were uniaxially pressed into pellets using a bench top Carver press (Carver Model C). Green bodies were prepared by pressing 0.3 g of milled powder in a 13 mm steel die well lubricated with stearic acid and acetone at a pressure of 150 MPa for 10 s. To improve the final green densities of the green bodies, uniaxially pressed pellets were further compacted through cold isostatic pressing. Uniaxially pressed pellets were vacuumed sealed in latex bags and then cold isostatic pressed at 210 MPa for 30 s.

Pellets were sintered at various temperatures (900 to 1300 °C for HA specimens and 700–1300 °C for MnHA specimens at 100 °C intervals) in air with a soaking time of one hour in a chamber furnace (CM, 1610FL, Bloomfield, NJ, USA). Specimens were heated at a ramp rate of 15 °C/min to the soaking temperature and then cooled to room temperature at a rate of 15 °C/min. A total of 10 pellets of HA or MnHA were sintered for each temperature point.

### 3.4. Characterization

The milled as-synthesized HA and MnHA powders were examined with a FEI Tecnai T12 S/TEM transmission electron microscope (TEM, FEI, Hillsboro, OR, USA). Powders were dispersed in ethanol via sonication and deposited onto a copper TEM grid with a carbon film.

As-synthesized HA and MnHA, along with sintered HA and MnHA specimens were assessed for phase purity using a Bruker D2 Phaser X-ray diffractometer (XRD, Bruker, Madison, WI, USA) with a copper target. Powder diffraction patterns were acquired over 2-theta ranging from 10–90° with a step size of 0.02° and a scan rate of 1.25 s per step.

Density measurements were performed on the final green bodies and sintered pellets using Ohaus digital balance with an accuracy of 1 mg and a caliper (Mitutoyo, Aurora, IL, USA) with an accuracy of 0.01 mm. Pellet height and diameter were measured in triplicates for each pellet and the average diameter and height were used to calculate the pellet density.

A Tinius Olsen (150 KS model) was used to determine the biaxial flexural strength on sintered 13 mm pellets using a 1000 N load cell at a crosshead speed of 0.01 mm/minute. A pin-on-disc fixture set up was used according to ASTM F 394 [61]. The modulus of rupture was calculated by the following equations:

(2) $$S=0.2387\frac{P\left(X-Y\right)}{d^{2}}$$

where *S* is the maximum center tensile stress (MPa) and *P* is the total load (N) causing fracture,

(3) $$X=\left(1+\nu \right)\ln\left[{\left(\frac{B}{C}\right)}^{2}\right]+\left(\frac{1-\nu }{2}\right){\left(\frac{B}{C}\right)}^{2}$$

(4) $$Y=\left(1+\nu \right)\left[1+\ln\left[{\left(\frac{A}{C}\right)}^{2}\right]\right]+\left(1-\nu \right){\left(\frac{A}{C}\right)}^{2}$$

where *ν* is Poisson’s ration, 0.27; *A* is the radius of the support circle (mm); *B* is the radius of the loaded area or pin tip (mm); *C* is the radius of the specimen and *d* is the specimen thickness at the point of fracture.

The microstructure of sintered HA and MnHA pellets was examined using a JEOL 6330F field emission scanning electron microscope (FESEM) operating at 5 kV. The fracture surface of sintered HA and MnHA pellets were sputter coated with gold palladium (SEM coating unit E5100, Polaron Instruments Inc., East Essex, UK) and used for microstructure analysis.

FESEM was also used to determine the grain size of HA pellets sintered over the temperature range of 1000 to 1300 °C. FESEM was used since the average grain size of certain specimens were less than the resolution limit of optical microscopy. Sintered pellets were polished to a 1 μm finish and then thermally etched for 30 min at 100 °C below the sintering temperature. The average grain size was determined from FESEM images using the line intercept method.

## 4. Conclusions

The sintering behavior of pure HA and MnHA was studied for the suitability of MnHA in load bearing applications. The maximum sintered density achieved for was 2.99 and 2.69 for HA and MnHA, respectively. Decomposition of MnHA into TCP and Mn_3_O_4_ occurred at 800 °C, while HA pellets started to sinter at 900 °C with partial decomposition to β-TCP and CaO. The maximum M.O.R achieved for HA and MnHA were 78 and 64 MPa, respectively. The low thermal stability of MnHA suggests that a faster dissolution rate *in vivo* than pure HA is expected.
